# Experiences and expectation with the use of health data: a qualitative interview study in primary care

**DOI:** 10.1186/s12875-022-01764-1

**Published:** 2022-06-23

**Authors:** Kadri Suija, Laura Alexandra Mardo, Reet Laidoja, Siim Nahkur, Anu Parvelo, Ruth Kalda

**Affiliations:** 1 Faculty of Medicine, Institute of Public Health and Family Medicine, University of Tartu, Tartu, Estonia; 2 Linnamõisa Family Practice Center, Tallinn, Estonia; 3 Estonian Family Physicians Association, Tallinn, Estonia; 4 Synbase OY, Tartu, Estonia

**Keywords:** Health data, Health information system, Primary care, COVID-19

## Abstract

**Background:**

Health data is important, however, not always well managed. The aim of this study was to investigate the experiences of patients and primary care physicians, their expectations and the obstacles encountered when using health data – both patient-generated as well as physician-generated.

**Methods:**

We conducted a qualitative interview study. We included adult persons who were ready to talk about the topic. Participants were recruited from primary care. The interviews were recorded using a dictaphone, transcribed verbatim and analysed using a content analysis method.

**Results:**

Altogether, we conducted 14 individual interviews, with patients (*n* = 7) and with physicians (*n* = 7). We found that both patients and physicians emphasized the importance of easy access to health data in digital health information systems. However, patients may not always understand medical terminology and physicians found that the quality of medical documents needs inspection. Both parties found that patient-generated data is a useful source of information, and that it should be used more often.

**Conclusions:**

The pandemic has highlighted the value of easy access to health data. The development of a health information system is useful to health care providers and patients, enables the transfer of evidence-based medicine, and supports health literacy.

## Background

The involvement of patients in their own care is one of the key issues faced in the management of chronic health problems and preventive work. It presumes a patient that is well-informed and possesses a high level of health literacy, as well as a positive collaboration between the patient and the health care provider, referred to as a person-centred approach [1]. Educating patients and supporting their self-management, as well as information technology development, are possibilities to increase person-centredness, but also to receive better health outcomes [2, 3].

Most of the health data is generated by physicians or other health care providers, although data generated by patients has recently been in much greater focus. Patient-generated health data can be defined as ‘health-related data, including health history, symptoms, biometric data, treatment history, lifestyle choices, and other information created, recorded, gathered, or inferred by or from patients or those who assist them to help address a health concern` [4]. Examples are blood glucose or blood pressure readings recorded with home health equipment; dietary/nutrition/exercise logs created, for example, using smartphone apps; questionnaires that patients complete and transmit to the care team prior to a face-to-face visit, etc. The quality and reliability of the data sources used by people vary, also there are considerations related to data privacy and security. All of this results in patient generated health data not being valued in the same way as physician-generated health data, meaning that its integration into the health information system is therefore limited [4]. On the other hand, questionnaires filled in by the patient in a quiet, comfortable room, or blood pressure measured in a home setting, may provide health care professionals with much more accurate information than the same activities performed in a busy and over-crowded health care centre or hospital setting.

During the pandemic, the use of phone and video consultations in health care grew exponentially, which also resulted in the need to evaluate the patient’s health and vital signs during a remote consultation. These circumstances placed a new perspective on the need to use patient-generated health data. Also, the tasks and the responsibility of the patient in managing their disease have increased [5].

Since 2008, Estonia has had a nationwide electronic health record system integrating data from different healthcare providers, creating a common record that patients are also able to access online through the Patient Portal [6]. However, due to technical reasons patients are unable to add health data, via questionnaire, diaries or other forms, into the Patient Portal, which means that the information exchange functionality is not being used.

The pace of progress in the digital world and technology development is rapid, which may put the education and healthcare system and the population in a weaker position. How to develop an up-to-date health information system and still keep the patient-doctor relationship in focus, how to maintain equity in health care, how to meet a challenge during new circumstances, e.g. health care during a COVID-19 pandemic, are the questions that need to be studied.

This study is part of the research project named “Development of Patient Report Questionnaire Prototype and Input Into Medical Documentation Standardization Process Based on COVID-19 Symptom Application” and included interviews with patients, physicians, and stakeholders. The aim of this substudy was to investigate the experiences of patients and primary care physicians, their expectations and the obstacles encountered when using health data – both patient-generated as well as physician-generated – during the COVID-19 pandemic.

## Methods

We conducted a qualitative interview study. The semi-structured interview guide was developed by the authors based on literature. Open questions were used for encouraging discussion and for enabling respondents to express their personal experiences. The topics covered in the interview guide are presented in Table 1.

**Table 1:** Interview topics

1. What do you think about questionnaires filled by the patients? By that we mean the questionnaire in which the person describes their medical issues independently prior to going to the doctor or the nurse.
2. What do you consider advantages regarding patient questionnaires?
3. What do you consider disadvantages regarding patient questionnaires?
4. Have you ever wished to present a paper, e.g. a blood pressure or nutrition diary, to your doctor or nurse? If so, how have you delivered it to them? Do you know how the paper was dealt with afterwards? OR Have your patients wanted to present you his/her blood pressure or nutrition diary or any questionnaire? If so, how have they delivered it to you?
5. What do you consider to be the best way to fill a patient questionnaire/diary? If needed, specify whether online or on paper, independently or with a doctor/nurse.
6. What do you think of the option of the patient questionnaire/diary being fillable on the Internet prior to the visit, e.g. through the patient portal?
7. What do you think about the option of getting instant feedback on further action after filling the questionnaire?
8. Do you think that patient questionnaires/diaries help you save time and/or money? If needed, specify the explanation and to whom the time and money belongs.
9. What is your opinion on medical documents on the whole, what should they be like? If needed, specify: the comprehensibility, safety, accessibility, practicality/usefulness of the documents.
10. Have you yourself read any of the health records regarding your doctor’s visits or investigation results through the patient portal? If so, was it easy to find and understand the document? If you have not, then what has prevented it (have not been to the doctor, have not been informed about it, did not find it, or other)? OR Do you use the health records in the central health information system during your clinical work? What do you think of the system in general?
11. Would you like to add anything else?

We used convenience sampling and included adults who were able to speak the Estonian language and who were ready to discuss the topic. Patients were recruited from two primary health care centres. In the case of physicians we first made a list of potential participants to find doctors with different backgrounds and age groups, and at the end of the interview we asked for suggestions from the interviewees for the next interview.

For participants who confirmed that they would like to participate, a convenient time and manner (phone or web platform) to conduct the interview was agreed upon.

All participants voluntarily signed the written informed consent to take part in the study before the interview. Participants were asked their age, gender and professional status.

The interviews were conducted by five researchers (SN, RL, AP, LM, or KS). Four of the researchers were medical doctors, and one has significant professional experience in conducting interviews; one researcher was male.

The interviews were recorded using a dictaphone and transcribed verbatim. We decided that we have enough participants based on the saturation of the data. We had regular discussion with the study team and in both groups (patients and physicians) after about fifth-sixth interview the answers were quite similar. We did not carry out any repeat interviews. The participants had possibility to comment the transcripts, but none of them used it.

The data was analysed using an inductive content analysis method [7–9]. Firstly, three researchers (KS, LM, and RK) read the transcripts to obtain an overview of the interviews and identified units of meaning, which were then categorized and labelled as codes. Secondly, the codes were sorted into groups sharing similar content. This was constantly compared and cross-referenced between transcripts. Thirdly, interpretations of the data were discussed between the researchers and interviewers to agree upon the broader themes. Unanimous consent was required from all of the authors prior to confirming a theme.

Study reporting was based on the COREQ criteria and recommended standards [10, 11].

The Research Ethics Committee of the University of Tartu approved this study.

## Results

### Participants

We conducted 14 semi-structured individual interviews: with patients (*n* = 7) and with physicians (*n* = 7) during the study period (March–May 2021). Most of the interviews (*n* = 11) were performed via web-based platform BigBlueButton and some (*n* = 3) by phone. In the patient group there were five women and two men, and in the physicians group there were four women and three men. The mean age of the subjects was 47.4 years for patients (29–81 years) and 38.57 years for physicians (26–65 years). Two of the patients were not working (due to maternity leave or retired). All physicians were working in the primary care, three of them were in the residency training.

The interviews were recorded using a dictaphone and lasted from 7 to 48 min, mean 25.96 min (physicians) and 16.56 min (patients).

### Themes

We identified three main themes in the interviews: 1) access to health records, 2) experience with using data in health records, and 3) the use of patient-generated data.

Data extracts are provided to illustrate these themes. The interviews were in Estonian language (native language of the interviewees). With the help of translator, who was native speaker of English, we translated the data into English during the last stage of the analysis. The quotes presented here are linked to participant age, gender (M/F), and study number.

### Access to health records

#### Patients

Patients find it important that they have access to their own health records (hospital epicrisis, consultation answers, investigation results, etc.). Most of the patients had used the central Patient Portal to review their health records. However, patients found that access to these documents via the Patient Portal is complicated.

“Anyway, everything is under a different name there, like epicrises or something like that. Maybe people just do not know what an epicrisis even is. That is the question, but yes, you can find them there, I have seen them.” (55, F, 10)

In the experience of patients, the health information system for patients should have a simple structure and should include all data related to a person’s health. It should give clear information and reminders about necessary health controls as well as vaccinations.

“As a patient, you want all the information regarding your health to be in one portal or platform and all clear, everything set out like your ears, your nose, vaccines /…/ So, for example, when you have a new tick vaccine date coming up, something will light up there and you will get a message or you will get an email.” (35, F, 12)

#### Physicians

Physicians find it important that all health data is consolidated in the central health information system, which functions efficiently and is also secure. In general, the security of the national e-health system is trusted.

“I do not see a real problem with safety here, because as long as we trust Estonia’s digital system and trust the X-Road and say that it is safe, I will believe that everyone’s health records are quite well protected.” (31, M, 24)

Physicians reported that easy access to previous patient health records is important to be able to prepare a complete treatment plan. Currently, the availability and usability of medical data from the central health information system is time consuming, due to the large amount of information, and it is poorly structured.

“I actually do quite a bit of work to prepare myself for patient appointments. I read those old epicrises. The time it takes varies /…/. It really depends on how much I need to read, if I really need a ten year summary, it will take a while, but it is also a matter of practice and I get better every time, but then I yet again discover myself having worked for 12 h. But it is very important to work through them.” (65, F, 26)

Interviewed physicians also mentioned that some medical epicrises (mostly from psychiatry) are unavailable in the central health information system, which makes it difficult to make correct medical decisions.

“The most confusing thing for me is why psychiatric records need to be restricted. This creates a lot of problems because we do not know the patient’s information. This is very important information – the patient’s mental side.” (24, F, 16)

The opinions of physicians in regards to the Patient Portal were inconsistent, with some doctors finding that patients having complete access to their health records could increase levels of anxiety, while others found that offering more information to patients is better than offering them less.

“I think that many patients would find the patient portal very interesting, but others would become too anxious or worried if they did not understand what those numbers in analysis results mean, for example. So they see that oh, I have a number here that is two units over the norm here, and think like, what am I going to do now, will I die.” (26, M, 25)

Physicians mentioned that the number of people interested in using the Patient Portal has increased during the COVID-19 pandemic, and even people who were not using it earlier, have now read or checked their health records.

“Now lately during corona it has been showing, of course, that they do have started to read their health records. This has not happened before, but I guess they are bored and have found it.” (65, F, 26)

### Experience with using data in health records

#### Patients

Patients mentioned that they expect medical records to give more information about disease, in general, as well as instructions for further self-care.

“People should have the kind of documents or information that would help them understand their illness or a syndrome, how they will live now, what they should do next, you know.” (52, M, 10)

Patients found it difficult to understand the medical abbreviations and language used by physicians in health records.

“I cannot understand it sometimes, especially with blood analyses, because I do not know what this or that abbreviation there means.” (47, F, 11)

Sometimes, they resorted to employing the help of other health care professionals in order to understand the medical documents, leaving them worried about how those whose acquaintances do not include doctors will manage.

“I myself have a doctor as a relative, I have that person, you know, so I have always gotten help with those, like, interpretations, so it has never been a big issue, but then I try to put myself in other people’s shoes, not that many people have that opportunity.” (29, M, 21)

In the opinion of patients, medical documents meant for use by health care professionals should be different from papers meant for patients.

“In that sense the document meant for a patient and the one meant for another doctor are two completely different things. Very often, they try to solve it with just one epicrisis, or actually, if we think about it, the doctor has to end up writing an epicrisis which is meant to be like a practical summary, and it is both to themselves and the patients, right.” (52, M, 10)

#### Physicians

Physicians were concerned about the inconsistent quality of medical papers (e.g. hospital epicrises, which sometimes only presented laboratory findings, but not a synthesis of the information and depend on the physician) as well as a clear management plan for the follow-up period (e.g. who is responsible for what) is often missing from the medical documents.

“And something that is definitely often missing is, who will then monitor the patient in the future and what should be the interval of monitoring and when would they return, that could be better worded, it is often left unclear.” (46, M, 27)

Physicians expect that structured text forms and more precise requirements (e.g. structure) for medical papers may help to harmonize the quality of documentations.

“If there were more forms and instructions for doctors, it would make the doctor’s work easier.” (28, F, 28)

In some interviews, physicians also mentioned the necessity for bigger changes to the health information system (e.g. the use of artificial intelligence, new document standards).

“If it is currently document based, then actually it should move towards being data based, and that data would be shown in the form that the user needs them in, and if we narrow it down, then, well, why do we even need that data.” (50, F, 29)

### Use of patient-generated data

#### Patients

Interviewed patients found that they were ready to generate important data required for medical decisions. For example, questionnaires filled in before the appointment could help to systematize complaints and also save time during the consultation.

“I think that it is reasonable, it saves time for both sides and usually in this questionnaire people are more open and maybe, they will write things that they maybe would not remember at the spot.” (33,F, 14)

One problem that patients mentioned is that not all people are able to generate this data, especially elderly and fragile persons.

“Well, considering that the ones with larger problems are the elderly, there should definitely be the option of the, like, paper version of the questionnaire.” (47, F, 11)

#### Physicians

Physicians found that patient generated data is valuable, and they took it into account when making treatment decisions.

“It is very good, especially if you are someone with blood pressure issues – you look at the blood pressure values, it fluctuates on some days, but you will know the average value and on that you will know whether the treatment is working or needs changing.” (26, M, 25)

It may give another value for a professional and broader view of the patient. They also believed that patients are eager to collect and present their collected health data to physicians.

“People sometimes make premature connections between some things, e.g. with some kinds of food and they restrict their diet. But if you monitor it, it gives the patient an overview and to the doctor as well, of course. Pain diaries are good too, for sure.” (28, F, 28)

According to the interviewed physicians, problems related to the use of patient-generated data involved the trustworthiness of the measurements collected by the patient and what to do with the written data they had recorded.

“We should think about whether there are some specific standards on which machines we trust and which we do not. So we should think it through so that the information would be valuable and not just plain noise.” (46, M, 27)

## Discussion

We found that both patients and physicians emphasized the importance of access to health data – physicians need it to make medical decisions, and patients are interested in their health in general. However, in order for such data availability and exchange to work better, it would be necessary to keep in mind that patients may not understand medical terminology. Also, physicians found that the quality of medical documents needs inspection. On the other hand, both parties found that patient-generated data is a useful source of information, and that it should be used more often.

Use of patient-generated data was mentioned often in our interviews. Patients were ready to collect their health data and doctors found that the measurements taken and questionnaires completed by people helped them in the diagnosis or treatment process. Similar findings are reported also in other studies [12]. Unfortunately, healthcare information systems do not always support the addition of patient generated data, by either the patient or caregiver, into the central electronic health record. Of course reliability is an important aspect as well. The latter is probably more important when it comes to using medical devices, such as in the measuring of blood pressure or blood sugar. Therefore, validated questionnaires filled in by patients are quite reliable. Moreover, some questionnaires, e.g. symptom-checkers could help, for example, in the triage process, which may save health care workers time [13] and reduce the burden of health care during a pandemic [14]. Thus symptom checkers have the potential to enhance the quality of care and healthcare system performance, which may also be employed during the documentation process.

Documentation is a legally and medically important part of a physician’s work. However, this is often also the most unsatisfying part of a physician’s work [15]. Quite often health information systems do not support the documentation process enough. This may explain why the quality of medical records is sometimes poor and some important aspects, like the management plan, may be missing. The latter was mentioned by the physicians in the current study.

There is some evidence that the use of scribes may reduce the amount of documentation [16]. The physicians interviewed in our study mentioned that forms and rules for documentations could harmonize the quality of health documents. A greater emphasis should also be placed on medical documentation during undergraduate and postgraduate education in medicine.

However, the improved documentation skills of doctors may not solve the problem of whether patients understand medical documents. Medical terminology is considered to be one of the most specialized and oldest terminologies in the world [17]. Thus the idea that data meant for use by medical professionals and common people must be presented in a different way, is still timely.

Interviewed patients in our study were interested in reading more about their disease, as well as self-care instructions from their medical documents stored in the health information system. This is of course a positive aspect that people are interested in their health and find medical papers to be necessary. Increasing the proportion of individuals who find their online medical records easy to understand is also one objective of the Health People 2030 initiative [3]. Moreover, it is proven that raising health literacy is related to achieving better health outcomes [3]. On the other hand, low health literacy is a comprehensive problem, one that escalated during the COVID-19 pandemic, as health care accessibility has decreased and uncertainty due to changes in the world have increased. Also, the physicians in the current study mentioned that patients have started to use Patient Portals more during the pandemic. There is a vast quantity of medical information available on the Internet; however, it is much more difficult to find reliable information. Primary care physicians, who are a first point of contact for most people, have particular place in educating patients and improving their health literacy [18]. It is likely that reading medical papers composed by familiar primary care physicians seemed a trustworthy source of information for patients, especially during the pandemic, if all health care is not easily accessible. According to the Dutch study, healthcare usage in primary care decreased 12% two years after the launch of an evidence-based health information website [19]. During the pandemic, a lot of new digital tools have been taken into use. Their impact will be explored in future studies. Hopefully, the development process will continue.

Health data security and trustworthiness is paramount. This aspect was mentioned by interviewed physicians but none of the patients reported concerns about privacy or the secure use of health data. However, security and privacy of health information cannot be taken for granted. More studies, how to improve health data security and how health care professionals can contribute in it, is needed.

### Limitations and strengths

The first strength of our study is that we included patients and physicians. Patients today are not only passive consumers in health care; therefore, it is important to collaborate with them. On the other hand, the role played by medical professionals in providing health data is still tremendous. The second strength is that we conducted our study in a primary care setting. Most of the people were treated in primary care centres, with only a minority requiring hospitalisation. The length of the interviews varied; interviews with patients were much shorter (mean time 16.56 min) than with professionals (mean time 25.96 min). Also, we discovered that there were more examples and greater variability among the answers provided by professionals than those of patients.

The limitations of our study are that the interviewed people were probably more interested in this topic. The latter could increase their eagerness to generate and read health data. Also, the pandemic has escalated the necessity of a progress in the digital health information system. The number of conducted interviews may seem small, but we achieved data saturation. We used convenience sampling because the time period was short and we did not had many prerequisites to participate in the study.

## Conclusions

The pandemic has highlighted the value of access to health data. The development of a health information system is useful to health care providers and patients, enables the transfer of evidence-based medicine, and supports health literacy.

## Data Availability

The datasets generated and analysed during the current study are not publicly available due to ethical reasons but are available from the corresponding author on reasonable request.

## Abbreviations

F: Female
M: Male
